# The Safety and Efficacy Profile of Magnesium-Based Bioresorbable Coronary Stents as Compared to Poly-L-Lactic Acid–Based Bioresorbable and Contemporary Drug-Eluting Coronary Stents—A Systematic Review

**DOI:** 10.1155/crp/7481956

**Published:** 2025-09-16

**Authors:** Liam Marsden Back, Aleksandra Gentry-Maharaj, Andrew Ladwiniec

**Affiliations:** ^1^Institute of Clinical Trials and Methodology, University College London, London, UK; ^2^Eastern Heart Clinic, Prince of Wales Hospital, Sydney, Australia; ^3^MRC Clinical Trials Unit at UCL, Institute of Clinical Trials and Methodology, University College London, London, UK; ^4^Department of Women's Cancer, Elizabeth Garrett Anderson Institute for Women's Health, University College London, London, UK; ^5^Cardiology Department, Glenfield Hospital, Leicester, UK

**Keywords:** bioresorbable, coronary, drug-eluting, magnesium, poly-L-lactic acid, stent

## Abstract

**Background:** Bioresorbable coronary stents (BRS) were designed with the aim of reducing the risk of late adverse events of permanent drug-eluting stents (DES) by dissolving once vessel patency had been restored and the requirement for acute mechanical support resolved. Bioresorbable poly-L-lactic acid (PLLA) scaffold designs, while initially appearing as promising technology, were unsuccessful in widespread clinical use due to an observed high rate of late stent thrombosis. Magnesium-based BRS (MgBRS) have provided an alternative to this original design and have shown promise in early-phase clinical trials. This review aims to address the clinical question: How does the current safety and efficacy evidence for MgBRS in all patients requiring percutaneous coronary intervention compare with the randomised data assessing PLLA-BRS and contemporary DES?

**Methods:** Two parallel systematic reviews were performed using the Preferred Reporting Items for Systematic Reviews and Meta-Analyses guidelines, utilising MEDLINE, EMBASE and Web of Science: the first assessing clinical outcomes of all observational and randomised MgBRS trials, the second assessing clinical outcomes of PLLA-BRS versus DES in randomised clinical trials. The primary safety and efficacy outcomes collected were cardiac death, target vessel failure (TVF) and stent thrombosis.

**Results:** A total of 3582 MgBRS patients (24 trials), 6370 PLLA-BRS and 5413 DES patients (16 trials) were included for analysis. Cardiac death was similar across all three stent designs in all time intervals. MgBRS performed similarly to contemporary DES and superiorly to PLLA-BRS at 12- and 24-month intervals with regard to TVF and stent thrombosis. Longer follow-up was suggestive of a poorer performance of MgBRS relative to DES, although with limited patient numbers.

**Conclusion:** MgBRS appear to perform similarly to DES and superiorly to PLLA-BRS at 12 and 24 months in regard to key clinical safety and efficacy measures. Further randomised studies are required before recommending this technology for widespread clinical use over DES.

## 1. Introduction

Despite growing sophistication in available medical therapies and procedures, coronary artery disease (CAD) remains the leading cause of mortality worldwide, contributing to 16% of total deaths occurring in 2019 [1]. CAD is characterised by the narrowing of a coronary artery due to atherosclerotic plaque formation within the vessel intima. This pathophysiologic process can precipitate both acute and chronic coronary syndromes with resulting myocardial ischaemia, infarctions, necrosis and death [2, 3]. These clinical syndromes prompted the development of mechanical technology to relieve this obstructive process via coronary angiography. This began initially in 1977 with a simple balloon inflation within an obstructive coronary lesion with no residual scaffolding left in situ [4]. While this novel intervention dramatically improved the treatment of CAD, high rates of vessel dissection and acute reocclusion post-procedure were observed [5]. Thus, the concept of a permanent implantable vascular scaffold or ‘stent' was derived, aiming to reduce the rates of recoil, acute closure and dissection following balloon angioplasty.

**Figure 1:**
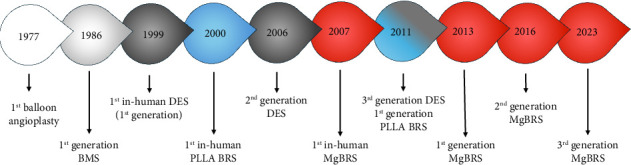
The timeline of development of coronary stent technology. BMS: bare metal stent; DES: drug-eluting stent; PLLA BRS: poly-L-lactic acid bioresorbable coronary stent; MgBRS: magnesium-based bioresorbable coronary stent.

The first coronary stent, implanted in 1986, revolutionised the treatment of CAD, and became the conception point for multiple subsequent iterations of coronary stent technology [5, 6] (Figure 1). The first devices inserted were a simple design, constructed from corrosion-resistant nonorganic metals only, predominantly stainless steel, and hence were named ‘bare metal stents' (BMS) [7]. These metallic compounds provided the necessary support and tensile strength to maintain vascular patency and device integrity; however, their permanence became the nidus for several future coronary complications [8–10].

To address some of these limitations of BMS, particularly neointimal hyperplasia, the technology of drug-eluting stents (DES) was conceptualised. This design involved a similar mechanical scaffold to the BMS generation, coated in a drug-eluting durable polymer that allowed localised elution of neointimal inhibiting drugs such as paclitaxel and sirolimus over the first few months following implantation [11–14]. While these coronary platforms were an improvement on previous BMS, they necessitated the use of prolonged dual antiplatelet therapy (DAPT) to avoid late stent thrombosis as a consequence of delayed epithelialisation of the drug-eluting durable polymer [15, 16]. These DES were subsequently refined to second and third generations, with improvements in metallic alloys, polymer and antiproliferative agents, in an aim to reduce risks of late stent thrombosis, neoatherosclerosis and chronic inflammation from the nondissolvable material [17–20]. This evolution of DES technology has drastically improved the prognosis of patients with obstructive CAD, with DES the uniformly recommended scaffold choice internationally for the contemporary interventional management of CAD [21].

## 2. Background

### 2.1. Rationale for Bioresorbable Technology and Failure of Poly-L-Lactic Acid (PLLA) Scaffolds

**Figure 2:**
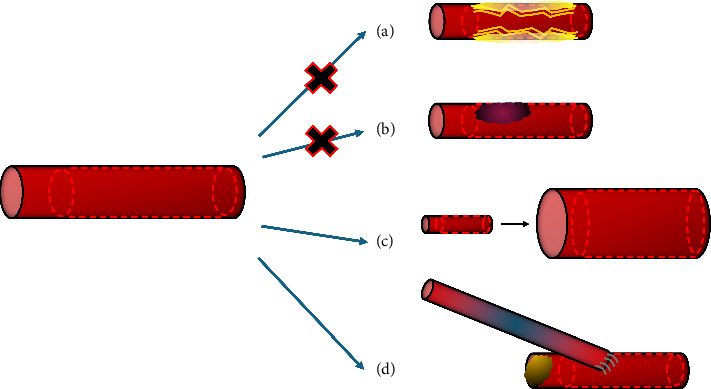
Proposed benefits of bioresorbable stent technology. (a) Minimise chronic local inflammatory reactions and subsequent neoatherosclerosis. (b) Remove nidus for stent thrombosis. (c) Allow for restoration of normal coronary vasomotion and demand vessel physiology. (d) Allow for future revascularisation procedures including coronary artery bypass grafting or repeat percutaneous intervention.

Despite continued improvements to the current third-generation technology, metallic DES are still limited by their permanent, rigid restraint of the affected vessel and the risk of intravascular complications as a consequence, primarily in-stent restenosis, neoatherosclerosis and stent thrombosis [22, 23]. With the aim of circumventing some of these limitations, research commenced into bioresorbable coronary stents (BRS) utilising both polymers and corrodible metals. It is hypothesised that a completely degradable coronary scaffold would reduce the risk of a local inflammatory reaction, minimise late stent thrombosis, as well as allowing the restoration of physiological coronary vasomotion [24–27]. Further theoretical advantages were proposed supporting this technology, particularly with regards to future intervention with coronary artery bypass grafting (CABG) if required [28] (Figure 2).

The first marketed BRS, the Absorb™ BRS (Abbott Vascular, Santa Clara, California, USA), utilised a degradable everolimus-eluting PLLA polymer [29, 30]. Unfortunately, following promising early results, the Absorb PLLA-BRS was withdrawn from clinical use in 2017 due to a significantly higher rate of device-related complications, particularly early and late stent thrombosis compared to DES [31–34]. This failure of technology is suspected to be multifactorial [35–37]. A larger diameter of stent strut in the PLLA design was required to achieve the same radial strength of a conventional metallic scaffold, resulting in more endothelial contact for chronic inflammation. Additionally, follow-up intravascular imaging studies demonstrated that this larger PLLA strut showed uneven and slower bioresorption than originally anticipated, leaving residual intravascular foreign material as a nidus for late thrombus formation when antiplatelet therapy was rationalised. Finally, liberal use of this stent technology in all patients and lesions post widespread release, including those with complex features such as significant calcification, tortuosity and thrombus, may have affected the PLLA-BRS performance in a real-world setting.

### 2.2. Benefits and Future Implications

#### 2.2.1. Magnesium-Based BRS

Given the device-related complications associated with the PLLA-BRS design, an increased interest developed in other bioresorbable platforms, particularly those magnesium-based, due to their favourable mechanical properties, unique biochemical features and established biocompatibility.

Magnesium-based implantable medical materials have long been known to have favourable biocompatibility, already used therapeutically for multiple indications including wound closure, dental implants and orthopaedic and cardiothoracic prostheses [38, 39]. The degradation profile of magnesium-based BRS (MgBRS) has been shown to be more rapid than PLLA-BRS designs, and when alloyed with zinc, aluminium or rare earth metals, demonstrates appropriately rapid bioresorption with maintenance of acute support required for obstructive CAD [40, 41].

**Figure 3:**
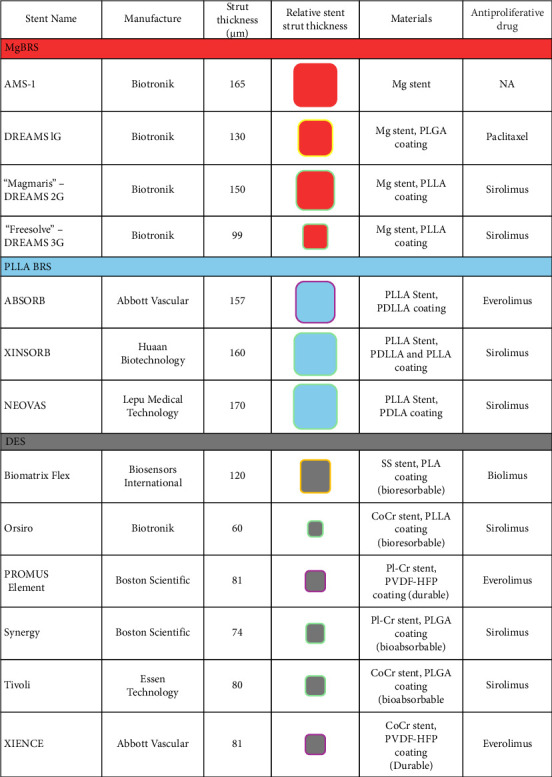
Stent characteristics from included trials. CoCr, cobalt-chromium; Mg, magnesium alloy; PDLLA, poly-D,L-lactic acid; Pl-Cr, platinum-chromium; PLGA, poly-lactic-co-glycolic acid; PLLA, poly-L-lactic acid; PVDF-HFP, poly(vinylidene fluoride-co-hexafluoropropylene); SS, stainless steel.

MgBRS have a superior mechanical profile to PLLA-BRS and have consistently achieved comparable radial strength to cobalt chromium and stainless-steel DES platforms in their original design, with no difference in the force required for longitudinal deformation between these stents [42]. Where PLLA-BRS have required thicker stent struts to achieve the relative radial strength of DES, maintenance of radial strength has been demonstrated in the current thin-walled MgBRS of 99 μm, a strut diameter approaching the values of contemporary DES [43] (Figure 3).

In the development of MgBRS, localised endothelial properties have been demonstrated with favourable implications for late coronary complications post implantation. Focussed magnesium concentration at the vessel intima appears to downregulate growth factor receptors and components of the extracellular matrix [44]. The result was a proliferation of coronary endothelial cells and quiescence of coronary smooth muscle cells, allowing for rapid endothelialisation and a reduction of neointimal formation within the MgBRS. Furthermore, in a comparison of acute thrombogenicity between MgBRS, PLLA-BRS and third-generation DES using an ex vivo porcine arteriovenous shunt model, MgBRS was found to have significantly less platelet adherence, thrombus deposition and inflammatory cell recruitment compared with PLLA-BRS, and performed similarly to third-generation DES [45].

## 3. Objectives

This systematic review aims to address the clinical question: How does the current safety and efficacy evidence for magnesium-based BRS in all patients requiring percutaneous coronary intervention compare with the randomised data assessing PLLA-based BRS and contemporary DES?

The availability of a safe, bioresorbable coronary scaffold for widespread use in obstructive CAD has the potential to provide an important option to mitigate some of the risks of long-term implanted durable material. MgBRS have been investigated in several, predominantly observational, clinical trials, as a potential favourable alternative to second- and third-generation DES. No large randomised controlled trials are as yet completed investigating this technology, and their use remains primarily in a clinical research setting. This study aims to critically appraise the available MgBRS literature, and identify areas required for further research.

## 4. Methods

This review was designed with the framework of the Preferred Reporting Items for Systematic Reviews and Meta-Analyses (PRISMA) guidelines [46]. This protocol is registered with the International Prospective Register of Systematic Reviews (PROSPERO), accessible at https://www.crd.york.ac.uk/prospero/ with registration number: CRD42024560168. No patient or public involvement was required in this study. All data collected was anonymous, and no data protection issues were identified. A network meta-analysis was provisionally considered, however, given the heterogeneity of included studies and patient populations, was deemed unsuitable.

### 4.1. Eligibility Criteria

To be eligible for inclusion in this systematic review, a study must have included individuals requiring percutaneous coronary intervention for CAD, with MgBRS, PLLA-BRS or DES. A publication was required to report the primary outcomes of interest, able to be extracted as incidence rates on an intention-to-treat (ITT) basis. Studies were still included for analysis if the secondary outcome was not performed or available.

### 4.2. Study Outcomes

The primary safety and efficacy outcomes collected are cardiac death, target vessel failure (TVF) and definite/probable scaffold thrombosis, at any time-point in available follow-up. TVF incorporates a composite of target vessel myocardial infarction or ischaemia-driven target lesion revascularisation. The secondary efficacy outcome of late-lumen loss, as assessed by quantitative coronary angiography (QCA) or intravascular imaging, was collected when available. These outcomes have previously been defined by the American Heart Association Academic Research Consortium on coronary device trials [47].

### 4.3. Search Strategy

Two parallel keyword systematic review searches were performed utilising databases MEDLINE, EMBASE, and Web of Science from 2007 to the 20^th^ June 2024. The date of 2007 was selected as this was the year of first publication of in-human implantation of MgBRS. All PLLA-BRS versus DES randomised controlled trials were published following this.

The first search addressing MgBRS utilised the following keywords:

- Magnesium OR Mg OR Magmaris OR Freesolve
- AND Bioresorbable OR Bioabsorbable OR Absorbable OR Dissolv∗
- AND Coronary (MESH).
- AND Stent∗ OR Scaffold∗ OR Vascular scaffold∗ (MESH)

All clinical data (including single-arm observational studies) were included for screening. Restrictions of human-only trials, published from 2007 to present were applied. Trade names of clinically studied MgBRS were included.

The second search addressing PLLA-BRS versus DES utilised the following keywords:

- Poly-L-lactic acid OR Polylactic acid OR PLLA OR Abbott Absorb OR Absorb
- AND Bioresorbable OR Bioabsorbable OR Absorbable Or Dissolv∗
- AND Coronary (MESH)
- AND Stent∗ OR Scaffold∗ OR Vascular scaffold∗ (MESH)

Restrictions of human-only trials, published from 2007 to present, and randomised controlled trials only, were applied. Trade names of the most widely studied PLLA-BRS were included.

**Figure 4:**
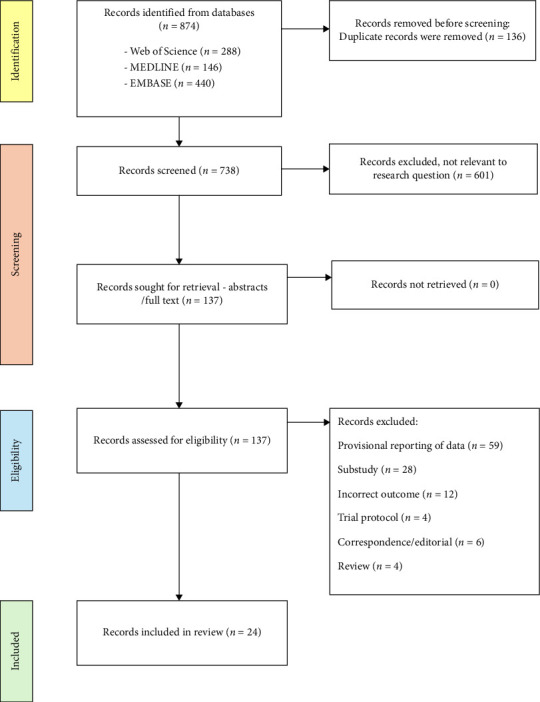
Magnesium-based bioresorbable coronary stents search strategy and study selection—completed using Preferred Reporting Items for Systematic Reviews and Meta-Analyses flow diagram.

**Figure 5:**
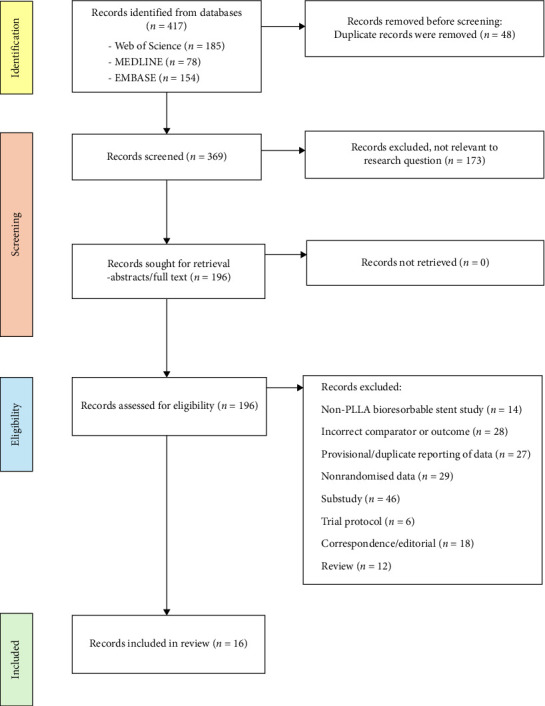
Poly-L-lactic acid versus contemporary drug-eluting stents search strategy and study selection—completed using Preferred Reporting Items for Systematic Reviews and Meta-Analyses flow diagram.

Duplicate articles were removed, and publications were screened by title and abstract for eligibility. Publications were subsequently retrieved for assessment. Reference lists of the included articles were further screened for missing publications. The details of the search strategy, screening and exclusions are summarised in two PRISMA flowcharts (Figures 4 and 5).

Data were extracted from the included studies and collated in a Microsoft Excel spreadsheet by the primary reviewer. Outcomes were evaluated at available follow-up durations and grouped for analysis into ≤ 12 months, 24 months, 36 months and 60 months. Where data were not available for these time periods, the field was marked as ‘not available' (NA). Results were presented as a grouped incidence. Late-lumen loss was infrequently reported in these trials. Where available, this was reported as ‘in-segment loss' in either cross-sectional diameter loss or minimal stent area (MSA) loss.

## 5. Results

The MgBRS search identified 874 publications for screening; 136 duplicates were removed, and a subsequent 601 excluded following review of title and abstract. A total of 137 publications were reviewed for eligibility for inclusion, of which 112 were excluded with reasons outlined in Figure 4. A total of 24 publications were assessed as eligible for inclusion in the MgBRS arm; 3 randomised controlled trials, 1 retrospective observational study and 20 prospective observational studies. These studies are summarised in Table 1. A total of 3582 patients receiving MgBRS with available outcome data were identified and included for assessment.

The PLLA versus DES search identified 417 publications for screening; 48 duplicates were removed, and a subsequent 173 excluded following review of title and abstract. A total of 196 publications were reviewed for eligibility for inclusion, of which 180 were excluded with reasons outlined in Figure 5. A total of 16 publications were assessed as eligible for inclusion in the PLLA versus DES arm, all randomised controlled trials. These studies are summarised in Table 2. A total of 6370 patients receiving PLLA-BRS and 5413 patients receiving contemporary DES with available outcome data were identified and included for assessment.

**Figure 6:**
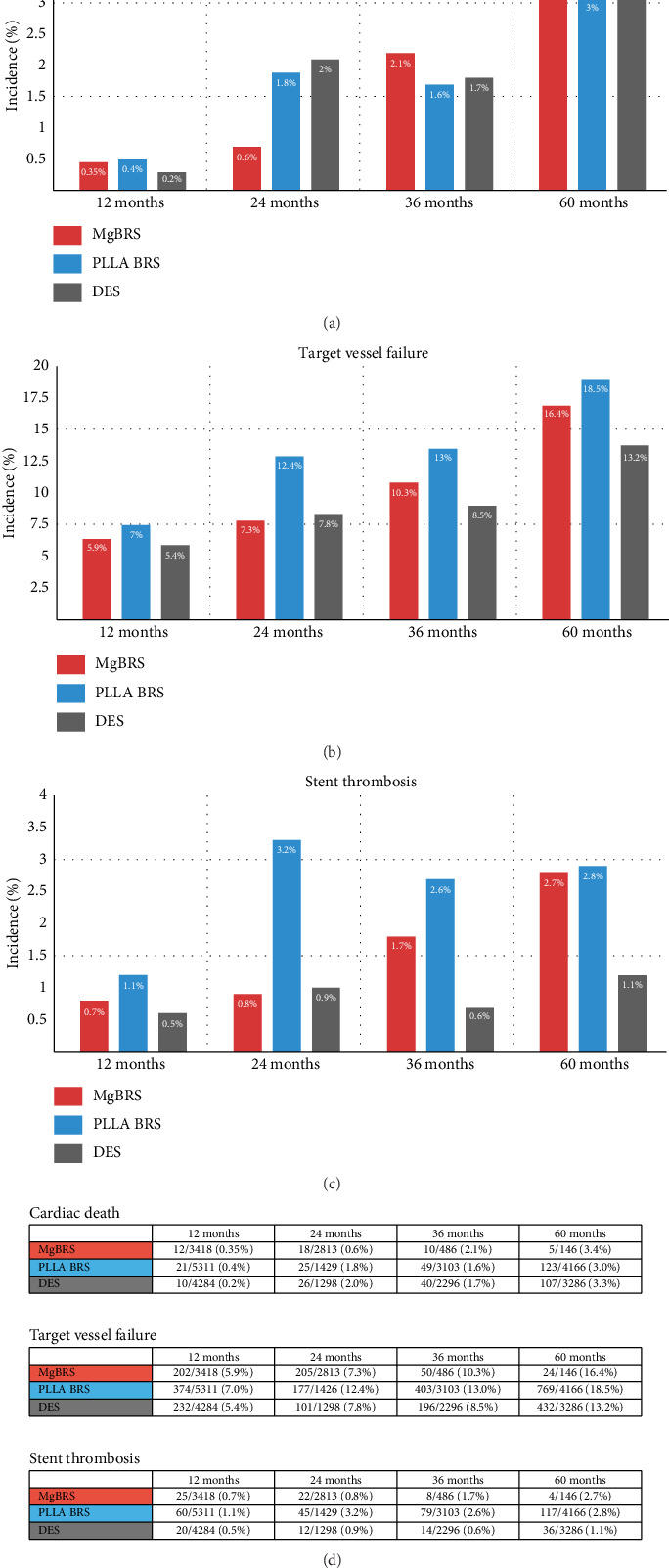
Primary outcomes: cardiac death (a), target vessel failure (b), stent thrombosis (c) and cumulative incidences (d) from included studies.

The pertinent characteristics of the included studies including patient presentation, lesion and relevant procedural characteristics are presented in Tables 3 and 4. The stents used in these studies are summarised in Figure 3. Clinical follow-up of all studies is summarised in Supporting Information 1A and 1B. All MgBRS studies excluding 1 reported outcome data ≤ 12 months. The patient pool in MgBRS studies beyond 24 months declined significantly in comparison to PLLA-BRS and DES, with only 412 and 146 patients available for analysis at 36 and 60 months, respectively. All PLLA-BRS versus DES trials excluding 2 reported outcome data ≤ 12 months, with all studies reporting outcomes ≤ 24 months. Long-term clinical outcome data were available in 3103 and 4166 PLLA-BRS patients at 36 and 60 months, respectively. Long-term clinical data were available in 2294 and 3286 DES patients at 36 and 60 months, respectively. Comparative late-lumen loss data were available in 6 MgBRS studies and 8 PLLA versus DES studies, with the majority in follow-up procedures ≤ 12 months. These results are summarised in Figure 6.

## 6. Risk of Bias Assessment

Quality assessment of the included studies was made using the Cochrane Risk of Bias tool for randomised studies (RoB 2), and Cochrane Risk of Bias tool for nonrandomised studies (ROBINS-1) [106, 107]. These findings are summarised in Supporting Information 2A and 2B.

The risk of bias in nonrandomised trials of MgBRS was variable, as anticipated from the heterogeneity of trial designs. In brief, most studies allowed proceduralists to utilise clinical experience when selecting patients for MgBRS implantation, and without specific criteria for those patients felt to be appropriate or inappropriate for inclusion. This has introduced a potential selection bias in those patients enroled for analysis, particularly with regards to inclusion of lower-risk or ‘simple' coronary lesions, which may not be applicable to a broader population. Although the necessity for crossover to conventional stenting with DES during the index MgBRS procedure was relatively rare, an excessive proportion (such as > 25% in BIFSORB PILOT-II) was occasionally observed. Finally, while clinical follow-up was excellent across the majority of trials (Supporting Information 1), the MAGMARIS ACS REGISTRY, BIFSORB PILOT-II and IT MASTERS REGISTRY reported < 80% clinical follow-up.

Risk of bias in PLLA-BRS versus DES trials was generally low across all included studies. Adequate study protocols could not be identified for COVER-AMI, PRAGUE-22, XINSORB and Hernandez et al. Randomisation procedures were not available for Hernandez et al.

These described aspects gave ‘some' concerns to the authors regarding risk of bias, although, for the purpose of this review, were not felt sufficient to exclude any individual publication from analysis.

## 7. Outcomes

### 7.1. Key Findings

Primary outcome data were available for all included studies. These primary outcomes for the collated data are presented in Figure 5. Raw datasets are available in Supporting Information 3 and 4.

### 7.2. Cardiac Death

Cardiac death across all three stent designs in all grouped periods was similar, with MgBRS performing equivalently to DES at 12 and 60 months, and superiorly at 24 months.

### 7.3. TVF

TVF was similar between MgBRS and DES at 12 and 24 months. The performance of PLLA-BRS appears poorer in TVF at all time points. At 60-month follow-up, the rates of MgBRS TVF approach that of PLLA-BRS (16.4% vs. 18.5%), suggesting a greater need for future revascularisation procedures in both BRS groups when compared to contemporary DES (13.2%).

### 7.4. Stent Thrombosis

In 12- and 24-month follow-up periods, MgBRS performed excellently with regards to stent thrombosis, with rates of only 0.7% and 0.8%, respectively. For comparison, DES showed stent thrombosis rates at 12 and 24 months of 0.5% and 0.9%, respectively. The significantly higher rate of early- and midterm stent thrombosis in PLLA-BRS compared to DES is again observed in this dataset, with PLLA-BRS stent thrombosis rates of 3.2% at 24 months. Although still performing superiorly to PLLA-BRS at 36 months, the limited 60-month follow-up of MgBRS (*n* = 146) shows stent thrombosis rates of 2.7% compared to DES rate of 1.1%.

### 7.5. Late Lumen Loss

Late lumen loss outcomes are presented in Table 5 and were variable across those studies in which they were reported. All studies described a degree of late lumen loss, regardless of quantitative measure. The observed trend of this data set suggests a worse performance in late lumen loss of MgBRS when compared to both PLLA-BRS and DES, however in limited, nonrandomised participants.

## 8. Discussion

As anticipated in this review's design, there is significant heterogeneity between trials in the MgBRS and the PLLA-BRS versus DES systematic reviews (Tables 1 and 2) and hence, is a primary limitation in the interpretation of these results.

Four different magnesium stent platforms were included in these three trials (Figure 3). The performance of the third MgBRS design, “Magmaris”, was investigated in 21 of 24 trials. Although only included in a small trial, the original MgBRS device, “DREAMS”, was designed without any drug-elution, a critical component of any contemporary stent. Unsurprisingly, the performance of MgBRS in this initial PROGRESS-AMS trial was poor in comparison to contemporary DES. These original MgBRS have been refined to the third-generation thinner-strut, drug-eluting design, “Freesolve”. This optimised design has only been studied in the BIOMAG trial to date, with excellent individual 12-month clinical outcomes (no cardiac death or stent thrombosis, 3.5% TVF) (Supporting Information 3), and offers a significant option for future research and clinical application.

In 10 of 16 trials of PLLA-BRS versus DES, only stable coronary syndromes were investigated, with only 4 targeting STEMI presentations. This is in contrast to the MgBRS trials, where 19 of 24 permitted NSTEMI or STEMI as an appropriate patient presentation. Additionally, the randomised PLLA-BRS trials have largely excluded high-risk patient and lesion characteristics, excluding the AIDA trial, designed to investigate PLLA-BRS in all-comer coronary presentations. These higher risk features such as heart failure, left main lesions and severe calcification were frequently included in the MgBRS studies. While this suggests that the MgBRS cohort may have been a more ‘complex' group, the nonrandomised design in addition to the ability of clinicians to exert discretion when selecting patients for enrolment introduces significant risk of selection bias.

Although still in a largely investigative phase of development, this systematic review reveals a large number of available patient clinical outcomes at 12 and 24 months of MgBRS, in addition to the known randomised data comparing PLLA-BRS and DES. While patient data remains comprehensive in the PLLA-BRS and DES groups out to 60 months, there is a significant drop off in long-term follow-up in the MgBRS, with only 486 and 146 patient outcomes available at 36 and 60 months, respectively. This raises some concerns about the accuracy and validity of the data when compared in these later time intervals.

Late lumen loss was difficult to compare between studies due to the heterogeneity of follow-up, preferred method of measurement and limited available studies. Previous DES research has suggested an angiographic late lumen loss of > 0.5 mm to be predictive of need for future TVF, with less than < 0.5 mm not being highly predictive [108]. All of the PLLA-BRS/DES trials meet these criteria. The results in the MgBRS trials are more variable, with Guiterrez-Baros et al. and the PRAGUE-22 study not meeting this criterion. The only study including the third-generation Freesolve MgBRS (BIOMAG-1), however, demonstrates a favourable lumen loss of 0.24 mm only. Interestingly, while it has been hypothesised that BRS technology would allow for positive remodelling following resorption of the vascular scaffold, this has not been well represented in either BRS group, with no late lumen gain demonstrated in any angiographic or intravascular imaging follow-up of BRS. Future trials should consider routine angiographic follow-up at > 36 months to increase our understanding of vascular remodelling post-scaffold absorption.

The use of intravascular imaging over the past decade has increased significantly in contemporary interventional cardiology practice, in line with multiple trials demonstrating its efficacy in reducing major adverse cardiac events [109, 110]. A criticism of the failure of PLLA-BRS technology is the underappreciation of complex lesion morphology and inadequate lesion preparation, with subsequent greater appreciation of the “4P's strategy” for lesion preparation. This can be considered with the use of intravascular imaging throughout this studied period. Of the PLLA-BRS/DES trials, only 9 of 16 reported the use of intravascular imaging, with a range of 0.4%–100%, however, the majority reporting < 30% use (Table 4). In the MgBRS trials, 20 of 25 reported on the use of intravascular imaging, with a range of 8.8%–100%, however, a majority reporting > 50% use (Table 3). Importantly, the BIOMAG study, the only study investigating the third-generation Freesolve MgBRS, reports a 97% penetrance of intravascular imaging use. Future MgBRS trials should mandate intravascular imaging at index procedure to optimise technique and minimise future coronary complications.

The most feared complication borne from the Absorb PLLA experience is that of late and very-late stent thrombosis [111]. In over 2800 patients, MgBRS has performed excellently in this safety outcome to 24 months. As recognised from PLLA experience, the frequency of stent thrombosis > 24 months is rare, consistent with the bioresorption of the polymer [96], and this is recognised in the plateau of stent thromboses in the longer-term PLLA-BRS data in this review. While MgBRS do reveal a signal of increased stent thrombosis in 36- and 60-month groups, it is recognised that this is a significantly smaller patient cohort than available for both PLLA-BRS and DES in the same period. DAPT is largely recommended for 12 months in the MgBRS trials, in an effort to avoid similar complications, particularly those more recently published. The earlier PLLA-BRS versus DES trial did not have uniform DAPT prescribing policies, and were often guided by recommendations for conventional DES. Given their unique biochemical profile, future use of MgBRS may mandate specific DAPT recommendations, separate from those utilised for DES guidelines, and provides an avenue for potential future research to optimise the safety profile.

## 9. Conclusion

BRS technology has the potential to significantly impact the risk of late complications secondary to durable implantable material. Pooled analysis of all available MgBRS data shows promising results for their safety and efficacy profile in comparison to the contemporary DES performance in the original PLLA-BRS randomised controlled trials. There is limited long-term follow-up data on the performance of MgBRS beyond 24 months, and a potential signal of increased TVF and stent thrombosis in the MgBRS arm when compared to DES. Due to the heterogeneous nature of the MgBRS technology used, as well as the differences observed in patient, lesion and technical selection between trials, these results are hypothesis generating only. Large randomised controlled trials with adequate short- and long-term follow-up, in addition to consideration for planned late angiographic assessments to investigate positive remodelling, are required before these stents can be recommended for general use.

## Figures and Tables

**Table 1:** Summary—magnesium-based bioresorbable coronary stent trials.

Study name	Year first published	Study type	Centres	Device type (MgBRS)	Comparator arm	Patients, *n*	Patients, *n*	Available outcome data		Follow-up duration (months)
						MgBRS	DES	Primary safety/efficacy	Secondary efficacy	
PROGRESS-AMS [48]	2007	Single arm, prospective	8	AMS	NA	63	NA	Y	N	4
BIOSOLVE-I [49, 50]	2013	Single arm, prospective	5	DREAMS 1G	NA	46	NA	Y	Y	1, 6, 12, 24, 36
BIOSOLVE-II [51–54]	2016	Single arm, prospective	13	MAGMARIS	NA	123	NA	Y	Y	6, 12, 36, 60
BIOSOLVE-III [55, 56]	2018	Single arm, prospective, observational	13	MAGMARIS	NA	61 (+123 biosolve II)	NA	Y	N	12, 36
BEST-MAG [57]	2018	Single arm, prospective, observational	1	MAGMARIS	NA	30	NA	Y	N	12
MAGMARIS ACS REGISTRY [58, 59]	2018	Single arm, prospective, observational	1	MAGMARIS	NA	193	NA	Y	N	1, 12, 24.
MAGSTEMI [60, 61]	2019	RCT	11	MAGMARIS	SES	74	76	Y	Y	12, 36
Ghafari et al. [62]	2019	Single arm, prospective, observational	1	MAGMARIS	NA	29	NA	Y	N	6
Carlier et al. [63]	2020	Single arm, prospective, observational	1	MAGMARIS	NA	35	NA	Y	N	6
BIOSOLVE-IV [64, 65]	2020	Single arm, prospective	80	MAGMARIS	NA	1075	NA	Y	N	12, 24, 36
CardioHULA REGISTRY [66]	2020	Single arm, prospective, observational	1	MAGMARIS	NA	42	NA	Y	N	12
Franze [67]	2021	Single arm, prospective, observational	10	MAGMARIS	NA	175	NA	Y	N	12
Gutiérrez‐Barrios et al. [68]	2021	Single arm, prospective, observational	5	MAGMARIS	NA	90	NA	Y	Y	6, 12, 24
BIFSORB Pilot II [69]	2021	Single arm, prospective, observational, proof-of-concept.	1	MAGMARIS	NA	20	NA	Y	N	1
PRAGUE-22 [70]	2021	RCT	2	MAGMARIS	DES	25	25	Y	Y	12
Fallesen et al. [71]	2022	RCT	1	MAGMARIS (OCT-guided implantation)	MAGMARIS (angiography-guided implantation	75	NA	Y	Y	6
MULTICENTRE Italian REGISTRY [72]	2022	Single arm, prospective, observational	4	MAGMARIS	NA	207	NA	Y	N	12, 24
Al Nooryani et al. [73]	2022	Single arm, prospective, observational	1	MAGMARIS	NA	54	NA	Y	N	30
Bossard et al. [74]	2022	Single arm, prospective, observational	1	MAGMARIS	NA	84	NA	Y	N	60
Truong et al. [75]	2023	Single arm, prospective, observational	1	MAGMARIS	NA	60	NA	Y	N	12
INTERNATIONAL MULTICENTRE DISCO REGISTRY [76]	2023	Single arm, retrospective cohort	26	MAGMARIS	NA	12	NA	Y	N	6
SHERPA MAGIC STUDY [77]	2023	Single arm, prospective, observational	18	MAGMARIS	NA	543	NA	Y	N	12
IT MASTERS REGISTRY [78]	2023	Single arm, prospective, observational	14	MAGMARIS	NA	350	NA	Y	N	12
BIOMAG-1 [79]	2023	Single arm, prospective	14	FREESOLVE	NA	116	NA	Y	N	6, 12

*Note:* Studies presented in chronological order of publication.

**Table 2:** Summary—poly-L-lactic acid bioresorbable coronary stents vs. drug-eluting stents trials.

Study name	Year first published	Study type	Centres	Device type	Comparator arm	Patients, *n*	Patients, *n*	Available outcome data		Follow-up duration (months)
						PLLA	DES	Primary safety/efficacy	Secondary efficacy	
ABSORB-II [29, 32, 80, 81]	2015	RCT	46	ABSORB	EES	335	166	Y	Y	12, 36, 48, 60
ABSORB-China [82, 83]	2015	RCT	24	ABSORB	EES	241	239	Y	N	12, 36, 60
ABSORB-Japan [84, 85]	2015	RCT	38	ABSORB	EES	266	134	Y	Y	12, 24, 60
de la Torre Hernandez et al. [86]	2016	RCT	1	ABSORB	EES	100	100	Y	N	12
STEMI-TROFI-II [87, 88]	2016	RCT	8	ABSORB	EES	95	96	Y	N	6, 36
Han et al. (Neovas) [89]	2017	RCT	32	NEOVAS	EES	278	282	Y	Y	12
EVERBIO-II [90]	2017	RCT	1	ABSORB	EES	80	160	Y	N	9, 12, 24
AIDA [91]	2017	RCT	5	ABSORB	EES	924	921	Y	N	12, 36, 60
ABSORB-III [92–94]	2017	RCT	193	ABSORB	EES	1322	686	Y	N	12, 36, 60
ABSORB-IV [95, 96]	2018	RCT	147	ABSORB	EES	1296	1308	Y	N	1, 12, 60
ISAR-ABSORB MI [97, 98]	2019	RCT	5	ABSORB	EES	173	89	Y	Y	6, 12, 24
COVER-AMI [99]	2019	RCT	1	ABSORB	EES	10	10	Y	N	3
Wu et al. (XINSORB) [100–102]	2020	RCT	2	XINSORB	SES	200	195	Y	N	12, 24, 36
COMPARE-ABSORB [103]	2020	RCT	45	ABSORB	EES	848	822	Y	N	12
Seo et al. [104]	2020	RCT	9	ABSORB	EES	171	170	Y	N	12
Eriksen et al. [105]	2022	RCT	1	ABSORB	EES	31	35	Y	Y	12

*Note:* Studies presented in chronological order of publication.

Abbreviations: EES = everolimus-eluting drug-eluting stent, RCT = randomised controlled trial, SES = sirolimus-eluting drug-eluting stent.

**Table 3:** Patient, lesion and procedure characteristics—magnesium-based bioresorbable coronary stent trials.

Study name	Patient characteristics			Inclusion criteria				Exclusion criteria					ACC/AHA lesion classification B2/C (%)	Intravascular imaging, %	Antiplatelet regimen
	Coronary syndrome	Age (mean ± SD)	Male (%)	De novo lesion	Reference vessel diameter (mm)	Lesion length (mm)	Lesion stenosis	LVEF < 30%/cardiogenic shock	Severe calcification	Left main	Bifurcation	Grafted vessel			
PROGRESS-AMS [48]	X	61.3 ± 9.5	70	De novo	X	X	X	X	X	X	X	X	X	IVUS, 100%	X
BIOSOLVE-I [49, 50]	Stable or unstable angina, or silent ischaemia.	65.3 ± 9.7	73.9	De novo	3–3.5	< 12	50%–99%	Excluded	Excluded	Excluded	Excluded	Excluded	X	OCT/IVUS, 93.5%	DAPT 12 months
BIOSOLVE-II [51–54]	Stable or unstable angina, or silent ischaemia.	65.2 ± 10.3	63	De novo	2.2–3.7	< 21	50%–99%	Excluded	Excluded	Excluded	Excluded	Excluded	44	OCT/IVUS, 24.3%	DAPT 6 months
BIOSOLVE-III [55, 56]	Stable or unstable angina, or silent ischaemia	65.5 ± 10.8	63	De novo	2.7–3.8	< 21	50%–99%	Excluded	Excluded	Excluded	Excluded	Excluded	61	X	DAPT 6 months
BEST-MAG [57]	STEMI	54.7 ± 10.2	76.7	All lesions	2.7–4.0	< 21	X	Excluded	Excluded	Excluded	Excluded	X	X	OCT/IVUS, 17%	Asprin/Ticagrelor 12 months
MAGMARIS ACS REGISTRY [58, 59]	Acute coronary syndrome including STEMI	66.3 ± 8.9	77.7	All lesions	2.7–3.7	< 21	50%–99%	Included	Excluded	Included	Included	X	26.6	OCT, 20%	DAPT 12 months
MAGSTEMI [60, 61]	STEMI	58.8 ± 10.6	85.1	De novo	2.75–3.75	All lesions	X	Included	Excluded	Included	Included	X	X	X	DAPT 12 months
Ghafari et al. [62]	All coronary presentations at clinicians' discretion	54 ± 60	75	De novo	X	X	X	X	X	X	X	X	X	OCT/IVUS, 8.8%	X
Carlier et al. [63]	All coronary syndromes at clinicians' discretion	55 ± 7	77	De novo	X	X	X	X	X	X	X	X	X	X	X
BIOSOLVE-IV [64, 65]	Symptomatic coronary disease excluding STEMI	61.3 ± 10.5	75	De novo	2.7–3.7	< 21	50%–99%	Excluded	Included	Excluded	Excluded	Excluded	15.2	X	DAPT 6 months
CardioHULA REGISTRY [66]	All coronary syndromes at clinicians' discretion	58.9 ± 11	85.7	De novo	2.5–3.5	All lesions	X	Excluded	Excluded	Excluded	Excluded	Excluded	X	OCT, 100%	DAPT, guideline per presentation
Franze [67]	All coronary syndromes at clinicians' discretion	55.5 ± 9	85.1	De novo	X	X	X	Excluded	Included	Included	Included	X	X	OCT/IVUS, 61.7%	X
Gutiérrez‐Barrios et al. [68]	Acute coronary syndrome including STEMI	55.9 ± 9.9	75.6	De novo	3.0–3.5	< 20	X	Excluded	Excluded	Excluded	Excluded	X	X	OCT, 68.9%	DAPT 12 months
BIFSORB Pilot II [69]	Stable angina, NSTEMI, or silent ischaemia	66 ± 10	82	De novo bifurcation lesion	X	X	X	Excluded	Excluded	Excluded	Included	X	X	OCT, 100%	DAPT, duration not reported
PRAGUE-22 [70]	Acute coronary syndrome including STEMI	57 ± 10.5	64	De novo	2.7–3.7	All lesions	X	Excluded	Excluded	Excluded	X	X	X	OCT, 92%	DAPT 12 months
Fallesen et al. [71]	NSTEMI	61.1 ± 10.9	79.9	De novo	2.75–4.0	< 28	> 50%	Included	Included	Included	Included	Included	44	OCT, 50%	Aspirin/Ticagrelor 12 months
MULTICENTRE Italian REGISTRY [72]	All coronary presentations at clinicians' discretion	60.8 ± 9.7	83	All lesions	All lesions	All lesions	X	Included	Recommended to avoid	Recommended to avoid	Excluded	Recommended to avoid	X	OCT/IVUS, 47%	DAPT 6 months minimum
Al Nooryani et al. [73]	All coronary presentations at clinicians' discretion	54 ± 11	85	All lesions	All lesions	All lesions	X	Excluded	Included	Included	Included	Included	X	OCT/IVUS, 80%	DAPT 12 months
Bossard et al. [74]	All coronary presentations at clinicians' discretion	62 ± 11	75	De novo	All lesions	All lesions	X	X	Included	Included	Included	Included	35	OCT/IVUS, 7%	DAPT 12 months
Truong et al. [75]	All coronary presentations at clinicians' discretion	59.4 ± 10.4	71	All lesions	< 3.75	All lesions	X	Excluded	Excluded	Excluded	X	X	45	IVUS, 98.3%	DAPT 12 months
INTERNATIONAL MULTICENTRE DISCO REGISTRY [76]	All spontaneous coronary artery dissection	50.9 ± 7.4	41.7	SCAD only	X	X	X	Included	Included	Included	Included	Included	Not reported	OCT/IVUS, 83%	X
SHERPA MAGIC STUDY [77]	All coronary presentations at clinicians' discretion	56.9 ± 9	78	All lesions	2.8–3.8	< 24	X	Included	Excluded	Excluded	Included	X	73	OCT/IVUS, 56%	DAPT 12 months
IT MASTERS REGISTRY [78]	Stable angina, NSTEMI, silent ischaemia.	59 ± 6	83	De novo	X	< 25	X	Included	Included	Included	Included	Included	61	X	X
BIOMAG-1 [79]	Symptomatic coronary disease excluded STEMI	61 ± 9	77.8	De novo	2.5–4.2	< 28	50%–99%	Excluded	Excluded	Excluded	Excluded	Excluded	76.9	OCT/IVUS, 97%	DAPT 12 months

*Note:* X; not reported or unavailable.

**Table 4:** Patient, lesion and procedure characteristics – poly-L-lactic acid–based bioresorbable coronary stent trials.

Study name	Patient characteristics			Inclusion criteria				Exclusion criteria					ACC/AHA lesion classification (%)	Intravascular imaging, %	Antiplatelet regimen
	Coronary syndrome	Age (mean ± SD)	Male (%)	De novo lesion	Reference vessel diameter (mm)	Lesion length (mm)	Lesion stenosis	LVEF < 30%/cardiogenic shock	Severe calcification	Left main	Bifurcation	Grafted vessel			
ABSORB-II [29, 32, 80, 81]	Stable angina, unstable angina, or silent ischaemia	61.5 ± 10	76	De novo	2.25–3.8	< 48	50%–99%	Excluded	Excluded	Excluded	Excluded	Excluded	46	IVUS, 100%	DAPT 12 months
ABSORB-China [82, 83]	Stable angina, unstable angina or silent ischaemia.	57.2 ± 11.4	71.8	De novo	2.5–3.75	< 24	50%–99%	Excluded	Excluded	Excluded	Excluded	Excluded	74.9	IVUS, 0.4%	DAPT 12 months
ABSORB-Japan [84, 85]	Stable angina, unstable angina or silent ischaemia	67.1 ± 9.4	78.9	De novo	2.5–3.75	< 24	50%–99%	Excluded	Excluded	Excluded	Excluded	Excluded	76	OCT/IVUS, 68.8%	DAPT 12 months
de la Torre Hernandez et al. [86]	Stable coronary disease	61.3 ± 12	76	De novo	> .5	X	X	Excluded	Excluded	Excluded	Excluded	X	Not reported	OCT/IVUS, 14.1%	X
STEMI-TROFI-II [87, 88]	STEMI	59.1 ± 10.7	76.8	All lesions	2.5–3.8	X	X	Excluded	Excluded	Excluded	Included	Excluded	Not reported	X	DAPT 12 months
Han et al. (Neovas) [89]	Stable coronary disease	58.5 ± 8.8	67.6	De novo	2.5–3.75	< 20	> 70%	Excluded	Excluded	Excluded	Excluded	Excluded	10.4	OCT, 28.2%	DAPT 12 months
EVERBIO-II [90]	Stable coronary disease	65	79	All lesions	X	X	X	Excluded	Included	Included	X	Included	Not reported	X	DAPT 6 months minimum
AIDA [91]	All coronary presentations at clinicians' discretion	64.3 ± 10.6	73	De novo	2.5–4.0	< 70	X	Included	Included	Included	Excluded	X	Not reported	X	DAPT 12 months
ABSORB-III [92–94]	Stable angina, unstable angina or silent ischaemia.	63.5 ± 10.6	70.7	De novo	2.5–3.75	< 24	50%–99%	Excluded	Excluded	Excluded	Excluded	Excluded	68.7	OCT/IVUS, 11.1%	DAPT, ‘as per standard practice'
ABSORB-IV [95, 96]	Stable angina, unstable angina or silent ischaemia.	63.1 ± 10.1	72	De novo	2.5–3.75	< 24	50%–99%	Excluded	Excluded	Excluded	Excluded	Excluded	47	OCT/IVUS, 14.2%	DAPT 12 months
ISAR-ABSORB MI [97, 98]	NSTEMI or STEMI with confirmed thrombus	61.7 ± 11	79.8	De novo	2.5–3.9	X	X	Included	Excluded	Excluded	Excluded	Included	Not reported	X	DAPT, ‘according to current guidelines'
COVER-AMI [99]	STEMI	56.5 ± 13.6	90	De novo	2.25–3.8	X	X	Excluded	Excluded	Included	Included	Excluded	Not reported	X	DAPT 12 months
Wu et al. (XINSORB) [100–102]	Stable coronary disease	60.2 ± 8.3	67.5	De novo	2.75–3.5	< 24	50%–99%	Excluded	Excluded	Excluded	Excluded	Excluded	4.3	X	DAPT 12 months
COMPARE-ABSORB [103]	All coronary presentations with high-risk for restenosis, excluding STEMI	62 ± 6	79.5	De novo	2.5–4.0	X	X	Excluded	Included	Excluded	Included	Excluded	Not reported	X	DAPT 12 months
Seo et al. [104]	All hemodynamically stable coronary presentations	63 ± 10	75	De novo	2.5–3.75	> 28	> 50%	Excluded	Included	Excluded	Excluded	Excluded	Not reported	OCT/IVUS, 49.3%	DAPT 6 months minimum
Eriksen et al. [105]	STEMI	60.7 ± 10.5	77.4	All lesions	X	X	X	Excluded	Excluded	Excluded	Excluded	X	Not reported	OCT, 100%	DAPT, duration not specified

*Note:* X, not reported or unavailable.

**Table 5:** Late lumen loss outcomes from included magnesium-based bioresorbable coronary stents, poly-L-lactic acid–based bioresorbable coronary stents, and drug-eluting stents, in included trials.

Study name	Qualitative method	Angiographic follow-up (% 12, 24, 36 m)	Late lumen loss, reported follow-up periods		
			≤ 12 months	≤ 24 months	≤ 36 months
MgBRS					
BIOSOLVE-I [49, 50]	QCA/IVUS	73.9%	0.39 ± 0.33 mm 1.3 ± 0.68 mm^2^ (MLA IVUS-guided)		
MAGSTEMI [60, 61]	QCA	87.8%	0.39 ± 0.49 mm		
Gutiérrez‐Barrios et al. [68]	QCA	36.7%	0.61 ± 0.75 mm		
PRAGUE-22 [70]	QCA/OCT	60%	0.54 ± 0.70 mm (QCA) 0.59 ± 0.37 mm (OCT)		
	
Fallesen et al. [71]	OCT	84%	2.3 ± 1.6 mm^2^ (MLA OCT-guided) 1.4 ± 1.4 mm^2^ (MLA angiography-guided)		
BIOMAG-1 [79]	QCA	86.0%	0.24 ± 0.36 mm		

BRS					
ABSORB-II [29, 32, 80, 81]	QCA/IVUS	NA, NA, 89%			0.37 ± 0.45 mm 0.56 ± 1.11 mm^2^ (MLA IVUS-guided)
ABSORB-China [82, 83]	QCA	86.3%	0.18 ± 0.03 mm		
ABSORB-Japan [84, 85]	QCA	95.6%, 89%	0.13 ± 0.30 mm	0.27 ± 0.38 mm	
STEMI-TROFI-II [87, 88]	QCA	90.5%	0.14 ± 0.28 mm		
Han et al. (Neovas) [89]	QCA	93.7%	0.14 ± 0.36 mm		
ISAR-ABSORB MI [97, 98]	QCA	81.3%	0.10 ± 0.39 mm		
Wu et al. (XINSORB) [100–102]	QCA	79.5%	0.19 ± 0.32 mm		
Eriksen et al. [105]	OCT	83.9%	2.06 ± 1.23 mm^2^ (MLA OCT-guided)		

DES					
ABSORB-II [29, 32, 80, 81]	QCA/IVUS	NA, NA, 91%			0.25 ± 0.25 mm 0.33 ± 0.97 mm^2^ (MLA IVUS-guided)
ABSORB-China [82, 83]	QCA	83.3%	0.13 ± 0.03 mm		
ABSORB-Japan [84, 85]	QCA	94.2%, 86%	0.12 ± 0.32 mm	0.12 ± 0.32 mm	
STEMI-TROFI-II [87, 88]	QCA	90.6%	0.06 ± 0.29 mm		
Han et al. (Neovas) [89]	QCA	89.9%	0.11 ± 0.34 mm		
ISAR-ABSORB MI [97, 98]	QCA	81.3%	0.10 ± 0.39 mm		
Wu et al. (XINSORB) [100–102]	QCA	77.8%	0.31 ± 0.41 mm		
Eriksen et al. [105]	OCT	65.7%	1.03 ± 1.15 mm^2^ (MLA OCT-guided)		

## Data Availability

The data that support the findings of this study are available in the article and the Supporting Information of this article.

## Nomenclature

AMS: Absorbable magnesium stent
BMS: Bare metal stent
BRS: Bioresorbable coronary stent
CABG: Coronary artery bypass grafting
CAD: Coronary artery disease
DAPT: Dual antiplatelet therapy
DES: Drug-eluting stent
DREAMS: Drug-eluting Absorbable Magnesium Stent
EES: Everolimus-eluting stent
ITT: Intention-to-treat
IVUS: Intravascular ultrasound
LLL: Late lumen loss
MESH: Medical Subject Headings
MgBRS: Magnesium-based bioresorbable coronary sent
OCT: Optical coherence tomography
PLLA: Poly-L-lactic acid
PLLA-BRS: Poly-L-lactic acid–based bioresorbable coronary stent
PRISMA: Preferred Reporting Items for Systematic Reviews and Meta-Analyses
QCA: Quantitative coronary angiography
RCT: Randomised controlled trial
RoB-2: Cochrane Risk of Bias Tools for Randomised Studies
ROBINS-1: Cochrane Risk of Bias Tools for Non-Randomised Studies
SES: Sirolimus-eluting stent
TVF: Target vessel failure

## Supporting Information

Supporting Information 1A: Clinical follow-up rates from included magnesium-based bioresorbable stent trials, categorised into 12 months, 24 months, 36 months and 60 months.

Supporting Information 1B: Clinical follow-up rates from included poly-L-lactic acid–based bioresorbable stent trials, categorised into 12 months, 24 months, 36 months and 60 months.

Supporting Information 2A: Risk of bias assessment for included magnesium-based bioresorbable stent trials using the Cochrane Risk of Bias tool for randomised studies (RoB 2) and Cochrane Risk of Bias tool for non-randomised studies (ROBINS-1).

Supporting Information 2B: Risk of bias assessment for included poly-L-lactic acid–based bioresorbable stent trials using the Cochrane Risk of Bias tool for randomised studies (RoB 2) and Cochrane Risk of Bias tool for non-randomised studies (ROBINS-1).

Supporting Information 3: Raw data set for primary and secondary outcomes for included magnesium-based bioresorbable coronary stent trials.

Supporting Information 4: Raw data set for primary and secondary outcomes for included poly-L-lactic acid–based bioresorbable coronary stent trials.
