# Supplementary material for: The Safety and Efficacy Profile of Magnesium-Based Bioresorbable Coronary Stents as Compared to Poly-L-Lactic Acid–Based Bioresorbable and Contemporary Drug-Eluting Coronary Stents—A Systematic Review
Source: Cardiol Res Pract. 2025 Sep 16;2025:7481956. doi: 10.1155/crp/7481956 (PMC12457060; doi:10.1155/crp/7481956)
Supplement: Supporting Information — Additional supporting information can be found online in the Supporting Information section. [file 7481956.f1.docx]

# **Supplemental Materials**

**Supplemental Material 1A –
Clinical follow-up rates from included magnesium-based bioresorbable stent trials**

| **Study Name** | **Follow Up** | | | |
| --- | --- | --- | --- | --- |
|  | **12 months** | **24 months** | **36 months** | **60 months** |
| **PROGRESS-AMS ^(56)^** | 100% |  |  |  |
| **BIOSOLVE-I ^(57, 58)^** | 93.5% |  |  |  |
| **BIOSOLVE-II ^(59-62)^** | 99% | 95% | 91% | 60% |
| **BIOSOLVE-III ^(63, 64)^** | 95.6% | 94% | 89.7% |  |
| **BEST-MAG ^(65)^** | 94% |  |  |  |
| **MAGMARIS ACS REGISTRY ^(66, 67)^** | 79% | 99% |  |  |
| **MAGSTEMI ^(68, 69)^** | 100% |  | 100% |  |
| **Ghafari et al ^(70)^** | 100% |  |  |  |
| **Carlier et al ^(71)^** | 100% |  |  |  |
| **BIOSOLVE-IV ^(72, 73)^** | 97.6% | 96.8% |  |  |
| **CardioHULA REGISTRY ^(74)^** | 100% |  |  |  |
| **Franze et al ^(75)^** | 100% |  |  |  |
| **Guitierrez-Barrios et al ^(76)^** | 100% |  |  |  |
| **BIFSORB Pilot II ^(77)^** | 63.6% |  |  |  |
| **PRAGUE-22 ^(78)^** | 100% |  |  |  |
| **Fallesen et al ^(79)^** | 84% |  |  |  |
| **MULTICENTRE ITALIAN REGISTRY ^(80)^** | 97.6% | 92.8% |  |  |
| **Al Nooryani et al ^(81)^** |  |  | 98% |  |
| **Bossard et al ^(82)^** | 95% | 94% | 89% | 86% |
| **Truong et al ^(83)^** | 100% |  |  |  |
| **INTERNATIONAL MULTICENTRE DISCO REGISTRY ^(84)^** | 100% |  |  |  |
| **SHERPA MAGIC STUDY ^(85)^** | 100% |  |  |  |
| **IT MASTERS REGISTRY ^(86)^** | 53% |  |  |  |
| **BIOMAG-1 ^(87)^** | 98.3% |  |  |  |

**Supplemental Material 1B –
Clinical follow-up rates from included Poly-L-Lactic acid based bioresorbable stent trials.**

| **Study Name** | **Follow Up** | | | |
| --- | --- | --- | --- | --- |
|  | **12 months** | **24 months** | **36 months** | **60 months** |
| **ABSORB-II ^(30, 33, 88, 89)^** | 98% |  | 93.4% | 76% |
| **ABSORB-CHINA ^(90, 91)^** | 99% |  | 98.1% |  |
| **ABSORB-JAPAN ^(92, 93)^** | 99.2% | 96.5% |  | 89% |
| **Hernandez et al ^(94)^** | 100% |  |  |  |
| **STEMI-TROFI-II ^(95, 96)^** | 90.6% | 99% | 98.4% |  |
| **Han et al (NEOVAS) ^(97)^** | 100% |  |  |  |
| **EVERBIO-II ^(98)^** | 100% | 97.5% |  |  |
| **AIDA ^(99)^** |  | 99.6% | 98.9% | 95.1% |
| **ABSORB-III ^(100-102)^** | 99.1% |  | 96.5% | 86.7% |
| **ABSORB-IV ^(55, 103)^** | 96.8% |  |  | 90.8% |
| **ISAR-ABSORB MI ^(104, 105)^** | 99.6% | 95.8% |  |  |
| **COVER-AMI ^(106)^** | 100% |  |  |  |
| **Wu et al (XINSORB) ^(107-109)^** | 93.9% |  | 94.1% |  |
| **COMPARE-ABSORB ^(110)^** | 97.5% |  |  |  |
| **Seo et al ^(111)^** | 97.4% |  |  |  |
| **Eriksen et al ^(112)^** | 89% |  |  |  |

**Supplemental Material 2A –
Risk of bias assessment for included magnesium-based bioresorbable stent trials.**

|  | **Randomisation Process** | **Selection of Participants** | **Classification of Interventions** | **Deviations from Intended Interventions** | **Missing Outcome Data** | **Outcome Measurement** | **Selection of the Reported Result** | **Overall Bias** |
| --- | --- | --- | --- | --- | --- | --- | --- | --- |
| **PROGRESS-AMS ^(56)^** |  | Some | Low | Some | Low | Low | Low | **Some** |
| **BIOSOLVE-I ^(57, 58)^** |  | Low | Low | Low | Low | Low | Low | **Low** |
| **BIOSOLVE-II ^(59-62)^** |  | Low | Low | Low | Low | Low | Low | **Low** |
| **BIOSOLVE-III ^(63, 64)^** |  | Low | Low | Low | Low | Low | Low | **Low** |
| **BEST-MAG ^(65)^** |  | Low | Low | Low | Low | Low | Low | **Low** |
| **MAGMARIS ACS REGISTRY ^(66, 67)^** |  | Low | Low | Low | Some | Low | Low | **Some** |
| **MAGSTEMI ^(68, 69)^** | Low | Low | Low | Low | Low | Low | Low | **Low** |
| **Ghafari et al ^(70)^** |  | Low | Low | Low | Low | Low | Low | **Low** |
| **Carlier et al ^(71)^** |  | Some | Low | Low | Low | Low | Low | **Some** |
| **BIOSOLVE-IV ^(72, 73)^** |  | Low | Low | Low | Low | Low | Low | **Low** |
| **CardioHULA REGISTRY ^(74)^** |  | Low | Low | Low | Low | Low | Low | **Low** |
| **Franze et al ^(75)^** |  | Some | Low | Low | Low | Low | Low | **Some** |
| **Guitierrez-Barrios et al ^(76)^** |  | Low | Low | Low | Low | Low | Low | **Low** |
| **BIFSORB Pilot II ^(77)^** |  | Some | Low | Some | Some | Low | Low | **Some** |
| **PRAGUE-22 ^(78)^** | Low | Low | Low | Low | Low | Low | Some | **Some** |
| **Fallesen et al ^(79)^** | Low | Some | Low | Low | Low | Low | Low | **Some** |
| **MULTICENTRE ITALIAN REGISTRY ^(80)^** |  | Low | Low | Low | Low | Low | Low | **Some** |
| **Al Nooryani et al ^(81)^** |  | Some | Low | Low | Low | Low | Low | **Some** |
| **Bossard et al ^(82)^** |  | Some | Low | Low | Low | Low | Low | **Some** |
| **Truong et al ^(83)^** |  | Low | Low | Low | Low | Low | Low | **Low** |
| **INTERNATIONAL MULTICENTRE DISCO REGISTRY ^(84)^** |  | Low | Low | Low | Low | Low | Low | **Low** |
| **SHERPA MAGIC STUDY ^(85)^** |  | Low | Low | Low | Low | Low | Low | **Low** |
| **IT MASTERS REGISTRY ^(86)^** |  | Low | Low | Low | Some | Low | Low | **Some** |
| **BIOMAG-1 ^(87)^** |  | Low | Low | Low | Low | Low | Low | **Low** |

**Supplemental Material 2B –
Risk of bias assessment for included poly-L-lactic acid-based bioresorbable stent trials**

|  | **Randomisation Process** | **Deviations from Intended Interventions** | **Missing Outcome Data** | **Outcome Measurement** | **Selection of the Reported Result** | **Overall Bias** |
| --- | --- | --- | --- | --- | --- | --- |
| **ABSORB-II ^(30, 33, 88, 89)^** | Low | Low | Low | Low | Low | **Low** |
| **ABSORB-CHINA ^(90, 91)^** | Low | Low | Low | Low | Low | **Low** |
| **ABSORB-JAPAN ^(92, 93)^** | Low | Low | Low | Low | Low | **Low** |
| **Hernandez et al ^(94)^** | Some | Low | Low | Low | Some | **Some** |
| **STEMI-TROFI-II ^(95, 96)^** | Low | Low | Low | Low | Low | **Low** |
| **Han et al (NEOVAS) ^(97)^** | Low | Low | Low | Low | Low | **Low** |
| **EVERBIO-II ^(98)^** | Low | Low | Low | Low | Low | **Low** |
| **AIDA ^(99)^** | Low | Low | Low | Low | Low | **Low** |
| **ABSORB-III ^(100-102)^** | Low | Low | Low | Low | Low | **Low** |
| **ABSORB-IV ^(55, 103)^** | Low | Low | Low | Low | Low | **Low** |
| **ISAR-ABSORB MI ^(104, 105)^** | Low | Low | Low | Low | Low | **Low** |
| **COVER-AMI ^(106)^** | Low | Low | Low | Low | Some | **Some** |
| **Wu et al (XINSORB) ^(107-109)^** | Low | Low | Low | Low | Some | **Some** |
| **COMPARE-ABSORB ^(110)^** | Low | Low | Low | Low | Low | **Low** |
| **Seo et al ^(111)^** | Low | Low | Low | Low | Low | **Low** |
| **Eriksen et al ^(112)^** | Low | Low | Some | Low | Low | **Some** |

**Supplemental Material 3 –
Raw data set for primary and secondary outcomes for included magnesium-based bioresorbable coronary stent trials.**

| **Study Name** | **Cardiac Death (n)** |  |  |  |
| --- | --- | --- | --- | --- |
|  | **Months** |  |  |  |
|  | **≤12** | **≤24** | **≤36** | **≤60** |
|  |  |  |  |  |
| **PROGRESS-AMS ^(56)^** | 0/63 | NA | NA | NA |
| **BIOSOLVE-I ^(57, 58)^** | 0/43 | NA | NA | NA |
| **BIOSOLVE-II ^(59-62)^** | 2/123 | 2/117 | 2/112 | 2/74 |
| **BIOSOLVE-III ^(63, 64)^** | 2/180 | 4/178 | 4/172 | NA |
| **BEST-MAG ^(65)^** | 0/30 |  |  |  |
| **MAGMARIS ACS REGISTRY ^(66, 67)^** | 0/153 | 0/191 | NA | NA |
| **MAGSTEMI ^(68, 69)^** | 1/74 | NA | 1/74 | NA |
| **Ghafari et al ^(70)^** | 1/29 | NA | NA | NA |
| **Carlier et al ^(71)^** | 1/35 | NA | NA | NA |
| **BIOSOLVE-IV ^(72, 73)^** | 2/1075 | 10/2057 | NA | NA |
| **CardioHULA REGISTRY ^(74)^** | 0/42 | NA | NA | NA |
| **Franze et al ^(75)^** | 1/175 | NA | NA | NA |
| **Guitierrez-Barrios et al ^(76)^** | 0/90 | NA | NA | NA |
| **BIFSORB Pilot II ^(77)^** | 0/11 | NA | NA | NA |
| **PRAGUE-22 ^(78)^** | 0/25 | NA | NA | NA |
| **Fallesen et al ^(79)^** | 0/75 | NA | NA | NA |
| **MULTICENTRE ITALIAN REGISTRY ^(80)^** | 0/202 | 0/192 | NA | NA |
| **Al Nooryani et al ^(81)^** | NA | NA | 0/53 | NA |
| **Bossard et al ^(82)^** | 1/80 | 2/78 | 3/75 | 3/72 |
| **Truong et al ^(83)^** | 0/58 | NA | NA | NA |
| **INTERNATIONAL MULTICENTRE DISCO REGISTRY ^(84)^** | 0/12 | NA | NA | NA |
| **SHERPA MAGIC STUDY ^(85)^** | 1/543 | NA | NA | NA |
| **IT MASTERS REGISTRY ^(86)^** | 0/186 | NA | NA | NA |
| **BIOMAG-1 ^(87)^** | 0/114 | NA | NA | NA |

| **Study Name** | **Target Vessel Failure (MI / Clinically Driven TVR)** |  |  |  |
| --- | --- | --- | --- | --- |
|  | **Months** |  |  |  |
|  | **≤12** | **≤24** | **≤36** | **≤60** |
|  |  |  |  |  |
| **PROGRESS-AMS ^(56)^** | 28/63 | NA | NA | NA |
| **BIOSOLVE-I ^(57, 58)^** | 3/43 | NA | NA | NA |
| **BIOSOLVE-II ^(59-62)^** | 6/123 | 6/117 | 6/112 | 8/74 |
| **BIOSOLVE-III ^(63, 64)^** | 4/180 | 6/178 | 7/172 | NA |
| **BEST-MAG ^(65)^** | 5/30 |  |  |  |
| **MAGMARIS ACS REGISTRY ^(66, 67)^** | 3/153 | 6/191 | NA | NA |
| **MAGSTEMI ^(68, 69)^** | 13/74 | NA | 14/74 | NA |
| **Ghafari et al ^(70)^** | 0/29 | NA | NA | NA |
| **Carlier et al ^(71)^** | 2/35 | NA | NA | NA |
| **BIOSOLVE-IV ^(72, 73)^** | 51/1075 | 154/2057 | NA | NA |
| **CardioHULA REGISTRY ^(74)^** | 2/42 | NA | NA | NA |
| **Franze et al ^(75)^** | 10/175 | NA | NA | NA |
| **Guitierrez-Barrios et al ^(76)^** | 10/90 | NA | NA | NA |
| **BIFSORB Pilot II ^(77)^** | 1/11 | NA | NA | NA |
| **PRAGUE-22 ^(78)^** | 3/25 | NA | NA | NA |
| **Fallesen et al ^(79)^** | 0/75 | NA | NA | NA |
| **MULTICENTRE ITALIAN REGISTRY ^(80)^** | 13/202 | 18/192 | NA | NA |
| **Al Nooryani et al ^(81)^** | NA | NA | 8/53 | NA |
| **Bossard et al ^(82)^** | 11/80 | 15/78 | 15/75 | 16/72 |
| **Truong et al ^(83)^** | 2/58 | NA | NA | NA |
| **INTERNATIONAL MULTICENTRE DISCO REGISTRY ^(84)^** | 1/12 | NA | NA | NA |
| **SHERPA MAGIC STUDY ^(85)^** | 32/543 | NA | NA | NA |
| **IT MASTERS REGISTRY ^(86)^** | 8/186 | NA | NA | NA |
| **BIOMAG-1 ^(87)^** | 4/116 | NA | NA | NA |

| **Study Name** | **Stent Thrombosis**  **(Definite or Probable)** |  |  |  |
| --- | --- | --- | --- | --- |
|  | **Months** |  |  |  |
|  | **≤12** | **≤24** | **≤36** | **≤60** |
|  |  |  |  |  |
| **PROGRESS-AMS ^(56)^** | 0/63 | NA | NA | NA |
| **BIOSOLVE-I ^(57, 58)^** | 0/43 | NA | NA | NA |
| **BIOSOLVE-II ^(59-62)^** | 0/123 | 0/117 | 0/112 | 0/74 |
| **BIOSOLVE-III ^(63, 64)^** | 1/180 | 0/178 | 0/172 | NA |
| **BEST-MAG ^(65)^** | 0/30 | NA | NA | NA |
| **MAGMARIS ACS REGISTRY ^(66, 67)^** | 0/153 | 0/191 | NA | NA |
| **MAGSTEMI ^(68, 69)^** | 2/74 | NA | 2/74 | NA |
| **Ghafari et al ^(70)^** | 0/29 | NA | NA | NA |
| **Carlier et al ^(71)^** | 1/35 | NA | NA | NA |
| **BIOSOLVE-IV ^(72, 73)^** | 5/1075 | 17/2057 | NA | NA |
| **CardioHULA REGISTRY ^(74)^** | 0/42 | NA | NA | NA |
| **Franze et al ^(75)^** | 2/175 | NA | NA | NA |
| **Guitierrez-Barrios et al ^(76)^** | 2/80 | NA | NA | NA |
| **BIFSORB Pilot II ^(77)^** | 0/11 | NA | NA | NA |
| **PRAGUE-22 ^(78)^** | 1/25 | NA | NA | NA |
| **Fallesen et al ^(79)^** | 0/75 | NA | NA | NA |
| **MULTICENTRE ITALIAN REGISTRY ^(80)^** | 1/207 | 1/192 | NA | NA |
| **Al Nooryani et al ^(81)^** | NA | NA | 2/53 | NA |
| **Bossard et al ^(82)^** | 4/80 | 4/78 | 4/75 | 4/72 |
| **Truong et al ^(83)^** | 1/58 | NA | NA | NA |
| **INTERNATIONAL MULTICENTRE DISCO REGISTRY ^(84)^** | 0/12 | NA | NA | NA |
| **SHERPA MAGIC STUDY ^(85)^** | 4/543 | NA | NA | NA |
| **IT MASTERS REGISTRY ^(86)^** | 1/186 | NA | NA | NA |
| **BIOMAG-1 ^(87)^** | 0/114 | NA | NA | NA |

| **Study Name** | **Late Lumen Loss** |  |  |  |
| --- | --- | --- | --- | --- |
|  | **Months** |  |  |  |
|  | **≤12** | **≤24** | **≤36** | **≤60** |
|  |  |  |  |  |
| **PROGRESS-AMS ^(56)^** | NA | NA | NA | NA |
| **BIOSOLVE-I ^(57, 58)^** | 0.39 +/- 0.33  N = 34/43 | NA | NA | NA |
| **BIOSOLVE-II ^(59-62)^** | NA | NA | NA | NA |
| **BIOSOLVE-III ^(63, 64)^** | NA | NA | NA | NA |
| **BEST-MAG ^(65)^** | NA | NA | NA | NA |
| **MAGMARIS ACS REGISTRY ^(66, 67)^** | NA | NA | NA | NA |
| **MAGSTEMI ^(68, 69)^** | 0.39mm +/- 0.49 | NA | NA | NA |
| **Ghafari et al ^(70)^** | NA | NA | NA | NA |
| **Carlier et al ^(71)^** | NA | NA | NA | NA |
| **BIOSOLVE-IV ^(72, 73)^** | NA | NA | NA | NA |
| **CardioHULA REGISTRY ^(74)^** | NA | NA | NA | NA |
| **Franze et al ^(75)^** | NA | NA | NA | NA |
| **Guitierrez-Barrios et al ^(76)^** | 0.61 +/- 0.75 | NA | NA | NA |
| **BIFSORB Pilot II ^(77)^** | NC | NA | NA | NA |
| **PRAGUE-22 ^(78)^** | 0.59 +/- 0.37mm | NA | NA | NA |
| **Fallesen et al ^(79)^** | -2.3+/-1.6mm2 (OCT) / -1.4+/-1.4mm2 (Angiography) | NA | NA | NA |
| **MULTICENTRE ITALIAN REGISTRY ^(80)^** | NA | NA | NA | NA |
| **Al Nooryani et al ^(81)^** | NA | NA | NA | NA |
| **Bossard et al ^(82)^** | NA | NA | NA | NA |
| **Truong et al ^(83)^** | NA | NA | NA | NA |
| **INTERNATIONAL MULTICENTRE DISCO REGISTRY ^(84)^** | NA | NA | NA | NA |
| **SHERPA MAGIC STUDY ^(85)^** | NA | NA | NA | NA |
| **IT MASTERS REGISTRY ^(86)^** | NA | NA | NA | NA |
| **BIOMAG-1 ^(87)^** | 0.24 +/- 0.36 | NA | NA | NA |

**Supplemental Material 4 –
Raw data set for primary and secondary outcomes for included poly-L-lactic acid based bioresorbable coronary stent trials.**

| **Study Name** | **Cardiac Death (n)** |  |  |  |  |  |  |  |
| --- | --- | --- | --- | --- | --- | --- | --- | --- |
|  | **BRS** |  |  |  | **DES** |  |  |  |
|  | **Months** |  |  |  |  |  |  |  |
|  | **≤12** | **≤24** | **≤36** | **≤60** | **<12** | **<24** | **<36** | **<60** |
|  |  |  |  |  |  |  |  |  |
| **ABSORB-II ^(30, 33, 88, 89)^** | 0/335 | NA | 3/325 | 5/256 | 0/166 | NA | 3/161 | 4/125 |
| **ABSORB-CHINA ^(90, 91)^** | 0/238 | NA | 1/236 | 1/236 | 3/237 | NA | 3/237 | 3/237 |
| **ABSORB-JAPAN ^(92, 93)^** | 0/265 | 1/261 | NA | 20/254 | 0/133 | 0/130 | NA | 8/127 |
| **Hernandez et al ^(94)^** | 1/100 | NA | NA | NA | 1/100 | NA | NA | NA |
| **STEMI-TROFI-II ^(95, 96)^** | 0/95 | NA | 2/96 | NA | 0/96 | NA | 0/96 | NA |
| **Han et al (NEOVAS) ^(97)^** | 1/278 | NA | NA | NA | 0/282 | NA | NA | NA |
| **EVERBIO-II ^(98)^** | NA | 1/80 | NA | NA | NA | 1/160 | NA | NA |
| **AIDA ^(99)^** | NA | 18/924 | 23/924 | 34/924 | NA | 23/921 | 26/921 | 41/921 |
| **ABSORB-III ^(100-102)^** | 8/1313 | NA | 18/1322 | 33/1322 | 1/677 | NA | 8/686 | 21/686 |
| **ABSORB-IV ^(55, 103)^** | 0/1254 | NA | NA | 30/1174 | 0/1272 | NA | NA | 30/1190 |
| **ISAR-ABSORB MI ^(104, 105)^** | 4/173 | 5/164 | NA | NA | 2/89 | 2/87 | NA | NA |
| **COVER-AMI ^(106)^** | 0/10 | NA | NA | NA | 0/10 | NA | NA | NA |
| **Wu et al (XINSORB) ^(107-109)^** | 1/200 | NA | 2/200 | NA | 0/195 | NA | 0/195 | NA |
| **COMPARE-ABSORB ^(110)^** | 5/848 | NA | NA | NA | 1/822 | NA | NA | NA |
| **Seo et al ^(111)^** | 0/171 | NA | NA | NA | 1/170 | NA | NA | NA |
| **Eriksen et al ^(112)^** | 1/31 | NA | NA | NA | 1/35 | NA | NA | NA |

| **Study Name** | **Target Vessel Failure (MI / Clinically Driven TVR)** |  |  |  |  |  |  |  |
| --- | --- | --- | --- | --- | --- | --- | --- | --- |
|  | **BRS** |  |  |  | **DES** |  |  |  |
|  | **Months** |  |  |  |  |  |  |  |
|  | **≤12** | **≤24** | **≤36** | **≤60** | **<12** | **<24** | **<36** | **<60** |
|  |  |  |  |  |  |  |  |  |
| **ABSORB-II ^(30, 33, 88, 89)^** | 19/335 | NA | 43/325 | 50/256 | 5/166 | NA | 5/161 | 9/125 |
| **ABSORB-CHINA ^(90, 91)^** | 10/238 | NA | 16/236 | 16/236 | 7/237 | NA | 8/237 | 8/237 |
| **ABSORB-JAPAN ^(92, 93)^** | 22/265 | 37/261 | NA | 51/254 | 8/133 | 11/130 | NA | 19/127 |
| **Hernandez et al ^(94)^** | 8/100 | NA | NA | NA | 5/100 | NA | NA | NA |
| **STEMI-TROFI-II ^(95, 96)^** | 1/95 | NA | 5/96 | NA | 0/96 | NA | 4/96 | NA |
| **Han et al (NEOVAS) ^(97)^** | 12/278 | NA | NA | NA | 10/282 | NA | NA | NA |
| **EVERBIO-II ^(98)^** | NA | 17/80 | NA | NA | NA | 8/160 | NA | NA |
| **AIDA ^(99)^** | NA | 108/924 | 125/924 | 159/924 | NA | 75/921 | 87/921 | 108/921 |
| **ABSORB-III ^(100-102)^** | 119/1313 | NA | 204/1322 | 248/1322 | 48/677 | NA | 79/686 | 100/686 |
| **ABSORB-IV ^(55, 103)^** | 98/1254 | NA | NA | 245/1174 | 82/1272 | NA | NA | 188/1190 |
| **ISAR-ABSORB MI ^(104, 105)^** | 9/173 | 15/164 | NA | NA | 6/89 | 7/87 | NA | NA |
| **COVER-AMI ^(106)^** | 3/10 | NA | NA | NA | 1/10 | NA | NA | NA |
| **Wu et al (XINSORB) ^(107-109)^** | 5/200 | NA | 10/200 | NA | 10/195 | NA | 13/195 | NA |
| **COMPARE-ABSORB ^(110)^** | 65/848 | NA | NA | NA | 46/822 | NA | NA | NA |
| **Seo et al ^(111)^** | 3/171 | NA | NA | NA | 4/170 | NA | NA | NA |
| **Eriksen et al ^(112)^** | 0/31 | NA | NA | NA | 0/35 | NA | NA | NA |

| **Study Name** | **Stent Thrombosis (Definite or Probable)** |  |  |  |  |  |  |  |
| --- | --- | --- | --- | --- | --- | --- | --- | --- |
|  | **Months** |  |  |  |  |  |  |  |
|  | **BRS** |  |  |  | **DES** |  |  |  |
|  | **≤12** | **≤24** | **≤36** | **≤60** | **<12** | **<24** | **<36** | **<60** |
|  |  |  |  |  |  |  |  |  |
| **ABSORB-II ^(30, 33, 88, 89)^** | 2/335 | NA | 9/325 | 9/256 | 0/166 | NA | 0/161 | 0/125 |
| **ABSORB-CHINA ^(90, 91)^** | 1/238 | NA | 2/236 | 3/236 | 0/237 | NA | 0/237 | 1/237 |
| **ABSORB-JAPAN ^(92, 93)^** | 4/265 | 8/261 | NA | 9/254 | 2/133 | 2/130 | NA | 2/127 |
| **Hernandez et al ^(94)^** | 0/100 | NA | NA | NA | 1/100 | NA | NA | NA |
| **STEMI-TROFI-II ^(95, 96)^** | 1/95 | NA | 2/96 |  | 0/96 | NA | 1/96 | NA |
| **Han et al (NEOVAS) ^(97)^** | 1/278 | NA | NA | NA | 0/282 | NA | NA | NA |
| **EVERBIO-II ^(98)^** | NA | 1/80 | NA | NA | NA | 0/160 | NA | NA |
| **AIDA ^(99)^** | NA | 31/924 | 34/924 | 43/924 | NA | 8/921 | 8/921 | 13/921 |
| **ABSORB-III ^(100-102)^** | 20/1313 | NA | 30/1322 | 32/1322 | 5/677 | NA | 5/686 | 7/686 |
| **ABSORB-IV ^(55, 103)^** | 9/1254 | NA | NA | 21/1174 | 4/1272 | NA | NA | 13/1190 |
| **ISAR-ABSORB MI ^(104, 105)^** | 3/173 | 5/164 | NA | NA | 2/89 | 2/87 | NA | NA |
| **COVER-AMI ^(106)^** | 0/10 | NA | NA | NA | 0/10 | NA | NA | NA |
| **Wu et al (XINSORB) ^(107-109)^** | 1/200 | NA | 2/200 | NA | 0/195 | NA | 0/195 | NA |
| **COMPARE-ABSORB ^(110)^** | 17/848 | NA | NA | NA | 5/822 | NA | NA | NA |
| **Seo et al ^(111)^** | 1/171 | NA | NA | NA | 1/170 | NA | NA | NA |
| **Eriksen et al ^(112)^** | 0/31 | NA | NA | NA | 0/35 | NA | NA | NA |

| **Study Name** | **Late Lumen Loss**  **(In-Segment)** |  |  |  |  |  |  |  |
| --- | --- | --- | --- | --- | --- | --- | --- | --- |
|  | **Months** |  |  |  |  |  |  |  |
|  | **BRS** |  |  |  | **DES** |  |  |  |
|  | **≤12** | **≤24** | **≤36** | **≤60** | **<12** | **<24** | **<36** | **<60** |
|  |  |  |  |  |  |  |  |  |
| **ABSORB-II ^(30, 33, 88, 89)^** | NA | NA | -0.29 +/- 0.46 | NA | NA | NA | -0.14 +/- 0.34 | NA |
| **ABSORB-CHINA ^(90, 91)^** | 0.18+/-0.03 | NA | NA | NA | 0.10+/-0.02 | NA | NA | NA |
| **ABSORB-JAPAN ^(92, 93)^** | 0.13 +/- 0.30 | 0.27 +/- 0.38 | NA | NA | 0.12 +/- 0.32 | 0.12 +/- 0.32 | NA | NA |
| **Hernandez et al ^(94)^** | NA | NA | NA | NA | NA | NA | NA | NA |
| **STEMI-TROFI-II ^(95, 96)^** | 0.14+/-0.28 | NA | NA | NA | 0.06+/-0.29 | NA | NA | NA |
| **Han et al (NEOVAS) ^(97)^** | 0.22 +/- 0.33 | NA | NA | NA | 0.16 +/- 0.28 | NA | NA | NA |
| **EVERBIO-II ^(98)^** | NA | NA | NA | NA | NA | NA | NA | NA |
| **AIDA ^(99)^** | NA | NA | NA | NA | NA | NA | NA | NA |
| **ABSORB-III ^(100-102)^** | NA | NA | NA | NA | NA | NA | NA | NA |
| **ABSORB-IV ^(55, 103)^** | NA | NA | NA | NA | NA | NA | NA | NA |
| **ISAR-ABSORB MI ^(104, 105)^** | 0.10+/- 0.39 | NA | NA | NA | 0.10 +/- 0.39 | NA | NA | NA |
| **COVER-AMI ^(106)^** | NA | NA | NA | NA | NA | NA | NA | NA |
| **Wu et al (XINSORB) ^(107-109)^** | 0.19+/- 0.32 | NA | NA | NA | 0.31 +/- 0.41 | NA | NA | NA |
| **COMPARE-ABSORB ^(110)^** | NA | NA | NA | NA | NA | NA | NA | NA |
| **Seo et al ^(111)^** | NA | NA | NA | NA | NA | NA | NA | NA |
| **Eriksen et al ^(112)^** | 1.73+/-0.92mm2 | NA | NA | NA | 1.16+/-1.03mm2 | NA | NA | NA |
